# Free-Text Responses in a Nationally Representative Experimental Survey about End-of-Life Care Choices: ChatGPT-4o-Assisted Qualitative Analytical Study

**DOI:** 10.2196/76335

**Published:** 2025-10-29

**Authors:** Elizabeth M Goldberg, Mario Macis, Megan Bounds, Jonathan Gomez Picazo, Lauren Hersch Nicholas

**Affiliations:** 1 Department of Emergency Medicine, CU Anschutz, 12631 East 17th Avenue 2nd Floor, Room 2519, Aurora, CO, 80045, United States, 1 (303) 724 4712; 2 Department of Economics and Management, Carey Business School, Johns Hopkins University, Baltimore, MD, United States; 3 Division of Geriatrics, Department of Medicine, CU Anschutz, Aurora, CO, United States; 4 Center for Bioethics and Humanities, CU Anschutz, Aurora, CO, United States

**Keywords:** Chatbot, geriatrics, comfort care, life-extending care, qualitative research, survey

## Abstract

**Background:**

Little is known about how surrogates make end-of-life care choices for patients who lack the ability to make decisions for themselves.

**Objective:**

The study aims (1) to identify key themes that emerged from participants’ free-text responses to a large nationally representative vignette survey about surrogate decision-making in end-of-life care and (2) to determine if an advanced artificial intelligence (AI) chatbot could assist us in accurately and efficiently performing qualitative analyses.

**Methods:**

Our dataset included 3931 free-text responses from a nationally representative survey of 6109 individuals. In this qualitative study, we first familiarized ourselves with the free-text responses and hand-coded the first 200 responses until we reached saturation. We then created a codebook, initial themes, subthemes, and illustrative quotes. Subsequently, we prompted ChatGPT-4o to analyze the entire dataset of 3931 responses and identify frequent keywords and generate themes and quotable quotes. We validated responses by comparing the AI’s keyword counts to qualitative software (NVivo, Lumivero) counts and cross-validating AI-generated quotes with the original transcripts.

**Results:**

We identified several key themes: surrogates more often chose comfort care for care recipients with dementia, particularly at advanced stages. They also strongly weighed the patients’ perceived quality of life and functional status. Many reported making surrogate decisions based on their own lived experiences or values, rather than making decisions aligned with the patients’ previously stated wishes. There was no significant difference between the AI and qualitative software’s keyword counts. The most frequent keywords included “life” (2051/81,713, 2.51%), “quality” (903/81,713, 1.11%), and dementia (507/81,713, 0.62%). Overall, AI-generated themes closely aligned with aforementioned human-generated themes. Manual coding of the first 200 free-text responses required 4 hours, including codebook development. In contrast, ChatGPT-4o generated themes in <10 seconds using the predefined codebook. However, dataset preparation, output verification, iterative prompting, debugging, and validation required several weeks.

**Conclusions:**

Surrogates often base end-of-life decisions on dementia stage, perceived quality of life, and their own lived experiences, rather than patient preferences. Using an AI chatbot to perform qualitative analysis on free-text responses may help extend the work of qualitatively trained investigators, especially for large datasets such as free-text responses to large surveys.

## Introduction

### Background

Many chronically ill older adults recognize the importance of discussing their end-of-life desires with their surrogates, family, friends, and clinicians, in the event they are unable to communicate during an emergency. Having advanced care directives reduces surrogate stress by avoiding the urgent decision-making process during emergencies and potential undesired treatment. It also may increase the likelihood that patients receive the care they desire, particularly in end-of-life situations where they may be unable to express their wishes [1]. Despite the increased use of advanced care directives, there are still incongruent care preferences between patients and their surrogates [2].

### Gaps and Preliminary Studies

This discordance in end-of-life decision-making suggests that better facilitation and understanding of end-of-life decision-making are needed [3]. Previous studies have focused on the effectiveness of advanced care directives, caregiver communication, or physician influence only [1,4-6]. However, the end-of-life decision-making process is complex, and individuals must consider multiple factors when deciding. Our team created a vignette-based cross-randomized survey to better understand surrogate factors in making end-of-life decisions [7]. Quantitative results suggest that surrogates were less likely to recommend life-sustaining treatment for persons living with dementia, that surrogates were more likely to ignore patient preferences for life-extending treatments when the person had dementia, and that surrogates were more likely to choose treatments that matched their own preferences when patients’ wishes were unclear.

### Objectives

In this study, our *objective* was to analyze free-text responses to the survey question “What were the main considerations that led you to give the answers you provided in the module above (which captured your end-of-life care choice for your care recipient)?” Given this survey included 6109 individuals, we decided to leverage artificial intelligence (AI) to assist our team in performing a qualitative analysis. Alternative existing approaches to analyzing such a large volume of data, including qualitative thematic analysis, have drawbacks including the need to select and analyze a smaller subset of responses to avoid the extensive resource use needed to perform traditional qualitative analysis [8]. Here, we share the findings of our qualitative analysis of free-text responses relating to end-of-life care decision making and detail how we used an AI-generative chatbot, ChatGPT-4o, to facilitate the analysis.

## Methods

### Data Sources

#### Overview

We analyzed free-text responses from a nationally representative, web-based experimental survey that included 6109 adults aged ≥18 years [7]. Thirty-five percent of survey respondents did not provide a free-text response to the question soliciting a rationale for the end-of-life care choice made in the vignette and thus were not included in this analysis. This survey was developed by co-authors LHN and MM. Both are PhD health economists and professors with experience studying the interaction between health care use and economic outcomes through combining survey, administrative, and clinical data. No relationship was established between the research team and participants before study commencement. A professional survey company, Ipsos Knowledge Network survey platform, recruited participants between December 8, 2022, and December 19, 2022, and ensured complete anonymity to the researchers, reducing social-desirability bias and other experimenter-demand concerns [9]. Surveys were available in English and Spanish, and participants were provided with devices to complete the survey if they lacked home computers or internet. This vignette-style survey was intended to systematically investigate surrogate decision-making in the context of dementia-related cognitive impairment and end-of-life care for older adults [7]. Further details on the type of data collected, timeline of data collection, and procedures are detailed in our previously published paper [7].

The objective of this paper was to analyze all the free-text responses qualitatively that were provided to the question “What were the main considerations that led you to give the answers you provided (Comfort Care or Life-extending Care) in the module above (which captured your end-of-life care choice for your care recipient)?” We followed the Standards for Reporting Qualitative Research (SRQR) guidelines (Checklist 1) in reporting this work [10]. Our dataset did not include any personal health information as this was an anonymous survey to a nationally representative group. Thus, we did not provide any personal health information to ChatGPT. While ChatGPT was used as a tool to perform efficient coding and theme generation, it was not used for writing assistance.

#### Summary

We performed several steps to conduct this qualitative analysis of free-text responses including (1) following traditional thematic analysis procedures to code and generate themes on a subset of free-text responses [11], (2) prompting ChatGPT-4o to use our codebook to generate its own themes and quotable quotes, and (3) debugging and validating the AI responses. We first familiarized ourselves with the data by reading and rereading the first 200 responses to the free-text question. Then, we hand-coded the first 200 responses, generating a preliminary code book and iteratively improving it through team discussion. Next, we created a list of preliminary themes and illustrative quotes following traditional thematic analysis procedures.

After providing the data and our codebook to ChatGPT-4o (GPT-4 variant, May 13, 2024; used via Google Chrome browser), we prompted it to generate relevant keywords, keyword counts, themes, and illustrative quotes. We performed several steps to validate the AI-generated results, including comparing AI-generated themes and quotes with themes and quotes generated by qualitatively trained human professionals and comparing keyword counts with counts generated by a commonly used qualitative analytic software, NVivo (Lumivero) [12]. Finally, we put the findings into the context of the current literature.

### Phase 1: Generating a Valid Approach

Prior to initiating the analysis, we conducted a literature review to identify how AI had been used in previous qualitative research. In particular, our methods were shaped by studies by Nashwan and Abukhadijah [13] and Van Veen et al [14], who provided prompt examples. Only limited studies have used AI approaches for analyzing free-text responses, so we also compared ideas on how to validate our AI findings with other academic teams doing similar work. These discussions yielded several suggestions, including comparing themes side by side between AI and human-generated themes and doing a keyword count comparison with commonly used qualitative software.

### Phase 2: Familiarization With the Data and Hand Coding

First, a physician scientist with experience caring for patients at the end of life and graduate-level qualitative training (EMG) and two other qualitatively trained research staff members (MB and JGP) read the first 200 participant responses. Then, 2 team members (EMG and MB) inductively coded the responses to the first 200 responses together. At this point, we reached thematic saturation, whereby no new significant themes continued to emerge, and concluded manual coding [15,16]. During this session, the team members created a preliminary coding schema in which we identified 6 major codes and several subcodes relating to end-of-life decision-making (Table S1 in Multimedia Appendix 1). The codebook was then iteratively refined through group discussion. Then, a third team member independently coded the same 200 responses (JGP). Finally, the principal investigator of the study (LHN), who designed the survey and had performed a preliminary human qualitative analysis after reading a sample of the responses, provided additional suggestions on the codes from her analysis, which we incorporated into the final codebook after team discussion.

### Phase 3: Human-Identified Themes, Subthemes, and Illustrative Quotes

Once we felt confident that our codebook reflected the topics and scope of the data from the first 200 respondents, we generated themes and quotes that were illustrative of the major and minor themes. Investigator triangulation (multiple investigators with multiple areas of expertise) was used to establish the trustworthiness of our findings [17]. We compiled a table to display themes and quotes.

### Phase 4: AI Analyses

Next, we used ChatGPT-4o to extend our human-generated analysis by providing it with the entire dataset of 3931 responders to the free-text question. We also piloted other generative AI tools (Microsoft Copilot and ChatGPT-3) but found that ChatGPT-4o provided the most usable responses and allowed us to upload larger amounts of data. Before entering the dataset in ChatGPT, we cleaned the data by removing all nonresponses. Free text containing responses of “none,” “n/a,” “na,” “no,” “nothing,” “no answer,” or “…,” were removed from the dataset submitted to ChatGPT to limit error. These responses were instead coded as “0” to indicate no free text was available. Data were uploaded via a Microsoft Word document containing a table with headings labeled “Free Text Responses,” “Age,” “Race/Ethnicity,” and “Gender.” ChatGPT processed the entire document at one time and required no chunking of information submission. No application programming interface or interface was used.

Our initial prompt to ChatGPT provided important context on the task, including that we planned to perform a qualitative analysis of free-text responses and that the AI should act as a qualitative analyst using our codebook and data to identify themes and quotable quotes. Although we considered having ChatGPT-4o inductively code the responses without human input, we anticipated that this would require much more investment by our team on the back end to iteratively prompt and refine themes. We also wanted to avoid the risk of irrelevant or erroneous themes or excessive AI “creativity.” We also prompted it to find the most common keywords in the text and generate keyword counts.

To illustrate how we completed this analysis, so it can be replicated, we share several sample prompts and how we validated responses:

1. The first prompt set up the research study context, its methods, and the AI’s role. An example prompt is: “This is a large survey study focused on end-of-life decisions, and you are a qualitative researcher analyzing the free-text responses to these questions.”
2. Next, we asked ChatGPT-4o to code the data using our codebook. A sample prompt includes: “This is a preliminary coding schema for our research. Using this codebook, code the entire sample. How did you do this?”
3. We asked ChatGPT-4o to create themes and subthemes and identify quotable quotes or key phrases that “make an impression on people from the text.” We then manually searched the dataset in NVivo to ensure the quote was verbatim and not fabricated.

Then, we asked the AI to create keywords identified in the entire dataset. We first quantified keywords by querying NVivo using the “word frequency/query” function. NVivo created a list of the most frequently used words in the dataset with the exception of proper names. NVivo provided the keyword, its frequency in the dataset, and the weighted percentage out of all words in the dataset. Then, we provided each keyword to ChatGPT-4o in addition to supplying it with a data file which included the full dataset of responses. We then queried ChatGPT-4o for exact keyword counts and weighted percentages, for example, “How often is the word ‘life’ found in this dataset, and what is the weighted percentage of ‘life’ among all words in the dataset?” by asking it to find how many times the specific keyword could be identified in the dataset.

### Ethical Considerations

The study was found to be exempt by the Johns Hopkins Homewood institutional review board (HIRB00012400), and we followed SRQR guidelines in reporting this work. Our dataset did not include any personal health information as this was an anonymous survey of a nationally representative group. Thus, we did not provide any personal health information to ChatGPT. The survey participants received compensation for their participation; the amount is unknown and proprietary due to a lump sum getting paid to Ipsos, the survey company.

## Results

### Overview

Free-text responses were created by 3931 (65%) surveyed participants. Free-text responders were significantly older, had higher educational attainment, were less likely to be working full time, and were more likely to be a caregiver for another adult than nonresponders. They were also significantly more likely to be married, have higher income, and carry a diagnosis of a chronic illness. Over half of free-text responders were people with chronic illnesses. A total of 1438 (37%) of them identified as caregivers, of which 601 (42%) stated that they provided ≥20 hours of care each week (Table 1). No significant differences were noted for sex, race, or region of the country.

**Table 1.** Participant demographics by free-text responder status.

Characteristics	Answered free-text question	
	No answers (n=2088)	Answer provided (n=3931)
Age (y), mean (SD)	51.19 (16.92)	55.08 (16.78)
Sex, n (%)		
Male	1016 (48.70)	1898 (48.28)
Female	1072 (51.34)	2033 (51.72)
Race, n (%)		
Black, non-Hispanic	229 (11.0)	400 (10.2)
Hispanic	103 (4.9)	148 (3.8)
Other, non-Hispanic	238 (11.4)	479 (12.2)
White, non-Hispanic	1449 (69.40)	2787 (70.90)
2+races, non-Hispanic	69 (3)	117 (3)
Education, n (%)		
No high school diploma or GED^a^	149 (7.1)	183 (4.7)
High school graduate (high school diploma)	652 (31.2)	963 (24.5)
Some college or associate degree	585 (28)	1099 (27.96)
Bachelor’s degree	392 (18.8)	882 (22.4)
Master’s degree or higher	310 (14.8)	804 (20.5)
Employment status, n (%)		
Working full time	980 (46.9)	1698 (43.20)
Working part time	277 (13.3)	553 (14.1)
Not working	831 (3.8)	1680 (42.74)
Marital status, n (%)		
Now married	1240 (59.39)	2418 (61.51)
Widowed	93 (4)	198 (5)
Divorced	207 (9.9)	424 (10.8)
Separated	31 (1.5)	63 (1.6)
Never married	517 (24.8)	828 (21.1)
Household income (US $), n (%)		
<10,000	98 (4.7)	135 (3.4)
10,000 to 24,999	205 (9.8)	319 (8.1)
25,000 to 49,999	371 (17.8)	645 (16.4)
50,000 to 74,999	345 (16.5)	657 (16.7)
75,000 to 99,999	247 (11.8)	501 (12.7)
100,000 to 149,999	384 (18.4)	702 (17.9)
≥150,000	438 (21)	972 (24.7)
Region of residence, n (%)		
Northeast	363 (17.4)	697 (17.7)
Midwest	486 (23.3)	873 (22.2)
South	787 (37.7)	1493 (37.98)
West	452 (21.6)	868 (22.1)
Caregiver for adult family member or friend, n (%)		
No	1504 (72.03)	2493 (63.4)
Yes	579 (27.8)	1438 (36.58)
Weekly hours spent caring for another adult, n (%)		
0-5	123 (21.8)	300 (20.9)
5-9	111 (19.6)	240 (16.8)
10-14	72 (13)	203 (14.2)
15-19	28 (5)	88 (6)
≥20	231 (40.9)	601 (15.3)
Chronic illness		
No	1066 (51.05)	1751 (44.54)
Yes	1022 (48.95)	2180 (55.46)

a GED: General Educational Development.

Human coding identified six major themes: (1) rationales provided for making end-of-life decisions, (2) the role of physical function in decision-making surrounding end-of-life care, (3) the role of dementia in decision-making surrounding end-of-life care, (4) impact on caregivers, (5) quality of life, and (6) basing decision on personal preferences or “I would” statements. Subthemes identified by human coding included: expected trajectory, drawing on individual experiences to decide, the caregiver feels a duty to choose to extend life, patient autonomy or preferences, ethics, emotional strain on family, limited quality of life with dependence on others, and living a life with value (Table 2).

**Table 2.** Human-derived major themes, subthemes, and quotable quotes.

Major theme and subtheme		Quotable quotes
Rationales provided for making end-of-life decisions		
Expected trajectory		*If there is no brain activity found then and no family to contest then make patient comfortable. Many things to be considered in all cases based on doctor advice and family concerns. All cases and circumstances are not the same! *[White non-Hispanic male with chronic disease, 72 y] *Mr. Jones is not able to eat, speak and move around. He would be in bed for the rest of his life, and that’s not how he should live his life. It’s very hard to be living under his conditions. *[Hispanic female adult, 54 y]
Drawing on personal experiences to decide		*My own mother has middle to late stage Alzheimer’s and I know her quality of life is already so impaired that it’s no way to live. She looks at a newspaper and asks if it contains the “numbers and directions for her”—she has trouble recognizing what a fork is. Alzheimer’s is heartbreaking and undignified for her. She is no longer who she was and does not know her own family. This is why I would choose for not invasive methods*. [White non-Hispanic female adult, 57 y]
A caregiver feels a duty to choose to extend life		*Even if the person can be kept alive, it’s not really living if the person is kept alive only to ease the guilt of a caregiver or significant other. I think the caregiver feels guilty if they do not try everything possible to extend life. *[White non-Hispanic female adult, 72 y]
Deciding based on patient autonomy and preferences		*She had decided before she became ill what she wanted so family wouldn’t have to decide that for her. I truly believe we should follow the patient’s wishes if written. *[Black non-Hispanic male caregiver, 73 y] *If a patient has a do not resuscitate order it should be followed, if not measures to sustain life [should] be used. *[White non-Hispanic male with chronic disease, 72 y] *Having had both parents in similar situations when in their 80s. Neither had an advanced directive, but me and sibs were clearly told for years from each that they did not want to live longer if bedridden.* [White, non-Hispanic male with chronic disease, 67 y]
Role of physical function in decision making surrounding end-of-life care		*Life has value, Mr. Smith has limited mobility but otherwise is fully aware of his own existence. I believe Mr. Smith wants to live! *[White non-Hispanic male adult, 61 y]
Role of dementia in decision making surrounding end-of-life care		*Mrs. Jones will never get better with the dementia. She will slowly lose her ability to talk and eat and the dementia will turn into Alzheimer’s disease, and she will pass away a horrible and painful death.* [White non-Hispanic female with chronic disease, 65 y] *She has dementia that will never improve and there is not much life after that diagnosis. In short there is very little quality of life and it will only get worse. *[White non-Hispanic male adult, 56 y] *He has dementia already and extending his life would not make his life any better!! *[White non-Hispanic female with chronic disease, 70 y]
Impact on caregivers		
Emotional strain on family		*I choose not to put the family through the pain of prolonging life that may not be beneficial and could hurt them more in the long run. *[Black non-Hispanic female adult, 55 y] *I know of this happening to a friend’s parent and it was an emotion[al] ride for both. Neither person had a good life until the parents passing*. [White non-Hispanic female adult, 67 y]
Quality of life		
Limited quality of life with dependence on others		*Quality of life is extremely important to me. Being dependent on others is not a good quality of life. *[White non-Hispanic female with chronic disease, 75 y] *If he is going to be treated with dignity, and respect, maybe he would have a half-way decent quality of life. Even if he isn’t aware of it. I would certainly hope so. *[White non-Hispanic female caregiver, 70 y]
Live a life with value		*If you can’t have quality of life your better off dying.* [White non-Hispanic female adult, 68 y]
Basing decision on personal preferences or “I would” statements		*If I was confined to bed for the rest of my life, I would choose comfort care if the opportunity arose.* [White non-Hispanic male with chronic disease, 31 y] *I put myself in those situations, primarily. Since I would not want unnecessary life extension, especially at 85+, I would not want this for anyone else. Interestingly, although I can fluctuate on my religious beliefs, the fact that I can consider an afterlife is comforting, and a reason to not artificially extend a few years here at the end. *[White non-Hispanic male caregiver, 68 y]

ChatGPT-4o identified 7 major themes from the free-text responses and also identified subthemes and representative quotes (Table 3). We further asked ChatGPT-4o to indicate the demographics of the responder (Table S2 in Multimedia Appendix 2) and indicate if the proxy was choosing comfort care, life-extending care, or both, which it performed accurately (Table S3 in Multimedia Appendix 3). In Table S3 in Multimedia Appendix 3, we also categorized errors made by the AI, including fabrication of quotes and substantial modifications of quotes. To aid in the replication of this work, we summarized the additional steps taken to ensure rigor in our analytic approach in Multimedia Appendix 4.

**Table 3.** ChatGPT-4o-derived themes using the codebook generated by humans.

Major theme and subtheme		Quotable quotes
Respect for advanced directives		
Honoring patient preferences		*The caregivers should follow the advanced directives. *[White non-Hispanic female, 51 y]
Clear directives prior to illness		*She said prior she did not want to have the invasive treatments. It is honoring her wishes. *[White non-Hispanic female, 67 y]
Quality of life		
Independence versus dependence		*If no quality of life, why take drastic measures to extend it? *[White non-Hispanic male, 88 y]
Burden of dementia		
Enjoyment of life and meaningful existence		*Life in bed is not a good life. Waste of resources that could be used on others. *[White non-Hispanic male, 75 y]
Loss of memory and cognitive decline		*With dementia, he lives in a state of confusion and fear. This will continue to get worse and quality of life will worsen even more. *[White non-Hispanic female, 54 y]
Emotional and financial toll on caregivers		*Having a caregiver 24-7 would cause an undue burden on family. Some family members would not be able to handle watching a family member deteriorate in front of their eyes.* [Black non-Hispanic male, 60 y]
Caregiver burden		
Emotional hardship for families		*The dementia has taken over, there is no quality of life. How much time are you buying to keep him in a state of no memory? *[White non-Hispanic female, 66 y]
Financial strain of prolonged care		*My father and my mother-in-law both had dementia, and it was very difficult and expensive to keep them alive. *[White non-Hispanic male, 62 y]
Ethical and religious considerations		
Sanctity of life		*Life, to all extents, is precious, given by a power greater than the limitations of medical care. *[White non-Hispanic male, 81 y]
Beliefs about life and death		*We are not the ones who should take away life. *[White non-Hispanic male, 87 y]
Patient dignity		
Maintaining respect during end-of-life care		*You should be able to die with dignity. Laying in bed is not living, it is existing. *[White non-Hispanic male, 72 y]
Avoiding perceived indignities		*End it so your son can get on with his own life. *[White non-Hispanic female, 75 y]
Decision-making challenges		
Lack of clear information		*It’s a tough call to make when all the information is just a few paragraphs. *[White non-Hispanic male, 56 y]
Emotional difficulty in making choices		*I am unable to respond because I don't believe I should decide on another person’s life.* [White non-Hispanic female, 83 y]

### Validation and Lessons Learned Using Generative AI

In Table 4, we depict a comparison of human- and ChatGPT-4o-generated major themes and subthemes. Several themes overlapped between human (black text) and AI (purple text) thematic analysis. While the AI did not always name the theme the same as our human analysts, the meaning was similar. Overall, there were several differences observed between human-generated and AI-generated tables, including that the AI closely relied on the codebook to generate themes, representative quotes were shorter, and more themes were generated by the AI. In addition, AI-generated themes were more descriptive—summarizing directly what participants said—rather than interpretative (eg, offering latent meaning or suggesting why participants may have said what was stated). ChatGPT created themes such as “quality of life” without extrapolating underlying motivations or cultural values, such as human-derived “drawing on individual experiences to decide.”

**Table 4.** Comparison of human-derived and artificial intelligence–derived thematic analysis.

Themes	Human-generated analysis	ChatGPT-4o analysis
Quality of life	✓	✓
Rationales provided for making end-of-life choices	✓	
Expected trajectory	✓	
Drawing on individual experiences to decide	✓	
Caregiver feels duty to choose to extend life	✓	
Deciding based on patient autonomy and preferences (respect for advanced directives)	✓	✓
Role of physical function in decision making surrounding end-of-life care	✓	
Role of dementia in decision making surrounding end-of-life care (burden of dementia)	✓	✓
Impact on caregivers (caregiver burden)	✓	✓
Basing decision on personal preferences or “I would” statements	✓	
Decision-making challenges		✓
Ethical and religious considerations		✓
Patient dignity		✓

We note that the AI generated 3 themes not included in our human-generated analysis: decision-making challenges, ethical and religious considerations, and patient dignity. While codes relevant to these topics existed in the human codebook, they were not prioritized in the final theme set due to their relatively lower frequency in the subset of responses reviewed manually. It is possible that these themes appeared more frequently across the full dataset analyzed by ChatGPT, which highlights the potential for AI methods to surface lower prevalence but potentially meaningful perspectives that might be missed in human review.

During the validation of ChatGPT-4o-generated quotable quotes by human analysts, we identified 3 major errors. First, ChatGPT-4o would often combine statements from multiple participants into one quote. Second, it sometimes ignored the prompt to create verbatim quotes and instead modified the quote to be more concise or grammatically correct. Third, it sometimes fabricated quotes entirely (hallucination). For the first error, combining statements, we addressed the challenge by reformatting the data into a table rather than using Microsoft Word, so the AI could more easily identify when one quote ended and another started. This improved ChatGPT-4o’s quotable quote reliability substantially. Our previous data format separated participant responses with spaces only. For the second error, paraphrasing, we reprompted ChatGPT-4o that exact quotes were needed, and they could not be modified. With reprompting, ChatGPT-4o was able to list verbatim quotable quotes. Below we display an example of the second error. While the meaning is the same, the content is paraphrased. For the third error, complete fabrication of quotes, we removed these from the tables and reprompted the AI to choose new verbatim quotes followed by verifying their existence in the dataset. We show more examples of all 3 types of errors in Table S3 in Multimedia Appendix 3.

Original quote by responder:

Life is precious and we should not try to end it of our own accord. It is God’s role to extend or end a life.

ChatGPT-4o-generated quote:

Life is precious and should be preserved as long as possible, according to God’s will.

In Table 5, we listed the 11 most common keywords identified by NVivo within the free-text responses and provided the counts and weighted percentages for keywords in NVivo and ChatGPT-4o. We chose the 11 most common keywords because starting at the 12th keyword, the frequency of the count was much lower. We identified that NVivo and ChatGPT-4o were using different methodologies to provide keyword counts and weighted percentages. Initial prompts to ChatGPT-4o generated counts and weighted percentages that were lower than NVivo, suggesting undercounting. We show how we corrected the discrepancy with the following example. To correct the count for the keyword “life,” we told ChatGPT-4o that NVivo had come up with a count of 2051 for the keyword “life” and asked it what it had done incorrectly, as its initial count for life was 1984. ChatGPT-4o responded that the NVivo count was correct, and what it was doing wrong was “The initial discrepancy was due to not properly accounting for cases where ‘life’ was followed by punctuation or appeared at the end of a sentence or line.” We then asked ChatGPT-4o to repeat the experiment using the correct method. Counts were still discrepant after this correction, though the ChatGPT-4o counts were closer overall to the NVivo counts after this reprompting. We then discovered that NVivo excluded “stop words,” which were considered insignificant. When we changed the NVivo settings to include the stop words, we then produced considerably closer weighted percentages, which are shared in Table 5.

**Table 5.** Keywords generated by NVivo compared to ChatGPT-4o-generated keywords and their frequencies.

Keyword	NVivo keyword count (weighted percentage), n (%)	ChatGPT-4o keyword count (weighted percentage), n (%)
Life	2051 (2.51)	2051 (2.48)
Quality	903 (1.11)	904 (1.09)
Care	551 (0.68)	551 (0.67)
Dementia	507 (0.62)	509 (0.61)
Want	481 (0.59)	481 (0.58)
Able	366 (0.45)	366 (0.44)
Live	366 (0.45)	366 (0.44)
Wishes	351 (0.43)	351 (0.42)
Family	346 (0.42)	346 (0.42)
Still	321 (0.39)	321 (0.39)
Think	255 (0.31)	255 (0.31)

### Time Considerations and Replication

Human coding was time-intensive; our team spent 4 hours coding the first 200 responses to the free-text question, recoding the first 200 responses, and creating and revising the initial codebook. ChatGPT-4o took <10 seconds to generate themes using our codebook. However, preparing the dataset for ChatGPT-4o, verifying the content, subsequent reprompting and verification, identifying methods for debugging, and validating took several weeks.

Due to ChatGPT-4o being a large language model (LLM), replication of similar outputs using the same prompt and dataset was challenging. We often received varying answers to the same command using ChatGPT-4o, although the overall major themes identified within the dataset remained relatively consistent. Once we reformatted responses into a table from the initial word file, ChatGPT-4o had more consistent response outputs to the same prompt.

## Discussion

### Principal Findings

In this ChatGPT-4o-assisted qualitative analysis, we identified several themes related to how surrogates make end-of-life care choices for patients. Proxies’ decisions are informed by prior caregivers’ prior lived experiences, expectations of quality of life, how advanced the stage of dementia is in the patient, and also personal values, including religious beliefs. In performing this analysis, we also learned several lessons about using AI tools to extend the capacity of our qualitative team. We learned that ChatGPT-4o is capable of using a human-created codebook to search free-text responses to create themes, representative quotes, and keywords. In addition, we learned that ChatGPT-4o-generated themes are primarily descriptive and closely match codes identified by humans. We also found that validation by humans is necessary, and ChatGPT-4o can sometimes provide insights on errors and correct them when prompted to do so.

This unique cross-randomized experiment allowed our team to provide new insights into what role dementia plays in surrogate decision-making. We found that many surrogates consider the stage of dementia to make end-of-life decisions. Caregivers may correctly identify that dementia is incurable and that extending life when dementia is advanced may not allow the individual to live the way they once did, free of the support of others. Others identified that quality of life in dementia may be tied to the ability to recognize and socialize with others, and when recognition diminishes, extending life may not be desired. Prior research also found that some surrogates equate dementia with loss of the “self” and view recognition as a key marker of dignity and quality of life [18]. Some felt extending life was warranted when the patient previously requested it or because of their religious beliefs.

Another common theme was drawing on individual experiences or preferences to make decisions. A key finding in the quantitative survey and the AI qualitative analysis was that when preferences were not clear, surrogates made choices that aligned with their own wishes. Personal beliefs about how one would feel if faced with these life choices were weighed in making the decision. However, ethically, proxies should make choices in line with patient preferences and not their own [19]. In some cases, proxies chose comfort care after projecting what *their* future self may feel like when aged or if *they* had the functional status of the patient described in the vignette. These survey and qualitative findings could inform clinical practice in several important ways: (1) clinicians should feel empowered to inform proxies that decisions should be made that are in line with patient preferences and (2) education should be provided to proxies about advanced dementia and its impact on quality of life, as well as the impact life-extending care may have on patient comfort and well-being.

Other literature on end-of-life choices and surrogate choices have found that while 90% of caregivers acknowledge that dementia is incurable, only 40% see it as life-limiting [20]. One study indicates that caregivers of people living with dementia who have chronic illnesses and who discuss goals of care with clinicians are more likely to have accurate prognostic understanding on the end-of-life care for persons living with dementia [21]. In addition, caregivers with better prognostic understanding were more likely to state a preference for comfort-focused care, and their care recipients were less likely to receive burdensome interventions and have greater comfort during the dying process. Studies have also recognized racial differences in advanced care planning, with African American persons reporting lower rates of advanced care planning completion (89% vs 73%) and lower preference for comfort care [22]. Several studies indicate that more education of proxies about the stages of dementia and end-of-life care options would be helpful to improve the quality of proxy decision-making [21,22].

The use of natural language processing AI-augmented qualitative analysis has been described as the “Way of the Future” [23]; however, few investigators have used it to perform qualitative analysis [24]. One team used structured topic modeling coupled with thematic analysis to analyze free-text data from nearly 38,000 English patrons with the aim to improve the user experience of public-facing services in the United Kingdom [25]. This team tested models with 5 to 40 topics and differing covariates and then had 2 human coders conduct thematic analysis to interpret the topics. In contrast with this approach, our approach did not require team members to have requisite skills in machine-learning methods and thus may be more accessible for qualitatively trained teams. Other AI approaches to analyzing large quantities of free text have included using natural language processing and machine learning models to develop robust classification models [26], but this approach also requires data science or related technical expertise.

Other teams have used ChatGPT-4o and more accessible LLMs to analyze qualitative data [23,27,28]. Experiences of these investigators can be summarized as overall positive in that these LLMs were found to be capable of producing satisfactory analyses that come close to human-generated analyses, but the performance of different LLMs varied, was not predictable, and serial prompting was necessary. Many have stated that themes are more descriptive than interpretive, yet it was easy to use and required less effort than manual coding. We also found that AI themes often did not go beyond what was explicitly stated by the participant and failed to explore latent meaning or social context. This could pose a barrier to using these tools in research where interpretative themes are essential. In addition, researchers found that even when using the same prompts and dataset, slightly different output was generated as AI generative chatbots “learn” over time. While this could improve performance, it also introduces problems with reproducibility. Thus, our team found it important to carefully log all prompts and responses in a log. In contrast to our approach, Carvalho et al [27] used a “series of chain-of-thought prompts to dig deeper into the provided response.” This approach had 2 advantages. It allowed the investigators to guide the chatbot to provide them with more positive themes when negative ones were initially suggested, and it surfaced less commonly encountered themes, allowing for more diversity of perspectives. Carvalho et al [27] also found that the paid version of ChatGPT-4o, which our team also used, generated the most accurate responses compared to the unpaid version and Google Gemini. This was particularly true when it was well-prompted and after multiple repetitions were performed in the same analysis.

Because there is currently no established gold standard for using advanced chatbots for qualitative analysis, our team made several deliberate methodological decisions. These included providing the AI a human-generated codebook rather than allowing it to inductively code responses and generate themes “independently.” Other teams may choose to allow the AI full discretion in its coding, and this approach could be more efficient and lead to more novel themes. We made this choice because our team had ample qualitative expertise, which allowed for us to code a subset of responses and ensure the codebook reflected key domains of interest. However, this approach may have reduced the AI’s ability to discover novel themes and constrained “creativity.”

Barriers to the use of AI in qualitative research include concerns over data privacy, a need for upskilling in AI among trained qualitative investigators, and a lack of established approaches to use the tool [24]. Information such as transcripts uploaded to AI platforms is used to develop the underlying algorithms that allow the computer to learn, which some academics feel is problematic from a data privacy standpoint. It is important for academics to follow institutional best practices such as obtaining approval from their ethical review boards prior to engaging in this work. Some institutions may advise using enterprise AI solutions or other tools that allow investigators to select that inputted data will not be externally shared or used to improve the AI’s algorithm. Another barrier has been that senior investigators trained in qualitative methods may not possess the skills to use AI for qualitative analysis or teach others how to use it. Similarly, a roadmap is not currently available that outlines how rigorous qualitative analysis can be done with AI assistance. We believe that AI will be increasingly used to analyze large qualitative datasets, and as its use and popularity grow, investigators must learn through trial and error how to best use it and debug it.

### Limitations

Free-text responses are an important source of data in surveys that collect quantitative measures because they can provide context to answer choices; however, unlike semistructured interviews, there is no opportunity to prompt the interviewee to elaborate on points or explain their statement when it is not clear. We found sentence fragments or short replies that made it difficult to understand what the surrogate’s perspective was and could be interpreted differently depending on the person reading the text. Although our research team has a multidisciplinary background, we recognize that we bring our own bias to our work, and this may have affected our interpretation of our findings. We made efforts to increase the validity of ChatGPT-4o’s analysis by asking it to show its work, comparing its output with existing qualitative software, and completing our own a priori human analysis on a subsample of the qualitative data, but the use of this tool for qualitative analysis is still new, and thus, there may be errors that we did not recognize.

### Conclusions

Participants in a vignette-style nationally representative survey asked to act as proxies provided several different rationales for why they chose to extend care or focus on comfort care in their hypothetical care recipients. Choices were often made based on whether the care recipient had dementia and how advanced it was, perceived quality of life, or the caregivers’ lived experiences or values, rather than making decisions aligned with the care recipients’ previously stated wishes. This suggests that it is in an individual’s best interest to share their care preferences with their intended proxy and engage in advance care planning prior to losing health care decision-making capacity.

Using an AI tool to perform qualitative analysis on free-text responses may help extend the work of qualitatively trained investigators, especially for large datasets such as free-text responses to large surveys. Our advice to investigators who aim to use ChatGPT-4o or other AI generative chatbots is to first complete a human-generated analysis using traditional qualitative coding, provide a codebook to the AI, anticipate the need for multiple prompts, try different LLMs, plan validation approaches and time for debugging, and keep a prompt and response log.

## Supplementary material

10.2196/76335 Multimedia Appendix 1 Preliminary coding scheme.

10.2196/76335 Multimedia Appendix 2 The themes, quotes, and demographic information produced by ChatGPT-4o after prompting demonstrate its ability to identify and match demographic information.

10.2196/76335 Multimedia Appendix 3 Initial artificial intelligence (AI)-generated themes by type of care (comfort care vs life-extending vs both) and human verification of AI-generated representative quotes with error type.

10.2196/76335 Multimedia Appendix 4 Additional method material to aid in replication of the study.

10.2196/76335 Checklist 1 SRQR checklist.

## Abbreviations

AI: artificial intelligence
LLM: large language model
SRQR: Standards for Reporting Qualitative Research
