# Supplementary material for: Free-Text Responses in a Nationally Representative Experimental Survey about End-of-Life Care Choices: ChatGPT-4o-Assisted Qualitative Analytical Study
Source: JMIR Aging. 2025 Oct 29;8:e76335. doi: 10.2196/76335 (PMC12571202; doi:10.2196/76335)
Supplement: Multimedia Appendix 1 [file aging-v8-e76335-s001.docx]

**SUPPLEMENT**

**Supplementary Table 1: Preliminary Coding Scheme**

| Theme: | Subtheme: |
| --- | --- |
| Advanced Directives/Preferences | Caregiver burden |
|  | Patient Dignity |
|  | Personal Relationships |
|  | Preference Stated (Full code, comfort, other) |
|  | Prolong Lifespan, Caregiver Duty |
|  | Patient Autonomy |
|  | Quantity and Quality of Information |
|  | Religious Beliefs and Ethics |
| Quality of Life | Is invasive treatment worth it? |
|  | Limited Quality of Life with Dependence on Others |
|  | Live a Life with Value |
| Caregiver Burden | Emotional Strain on Family |
| Dementia | Stages of Dementia |
| I would Terms, Personal Reactions | I (the proxy) wouldn’t live like this |
| Physical Function, mobility | Limited mobility but fine cognition |
