# Supplementary material for: Free-Text Responses in a Nationally Representative Experimental Survey about End-of-Life Care Choices: ChatGPT-4o-Assisted Qualitative Analytical Study
Source: JMIR Aging. 2025 Oct 29;8:e76335. doi: 10.2196/76335 (PMC12571202; doi:10.2196/76335)
Supplement: Multimedia Appendix 2 [file aging-v8-e76335-s002.docx]

**Supplementary Table 2: The themes, quotes, and demographic information produced by ChatGPT-4o after prompting demonstrating its ability to identify and match demographic information**

| Theme | Representative Quote | Age | Race/Ethnicity | Gender |
| --- | --- | --- | --- | --- |
| Quality of Life Considerations | "If no quality of life, why take drastic measures to extend it." | 88 | White, Non-Hispanic | Male |
|  | "Life in bed is not a good life. Waste of resources that could be used on others." | 75 | White, Non-Hispanic | Male |
| Respect for Patient Wishes | "His directive said to extend life." | 51 | White, Non-Hispanic | Male |
|  | "She said prior she did not want to have the invasive treatments. It is honoring her wishes." | 67 | White, Non-Hispanic | Female |
| Impact on Family | "Having a caregiver 24-7 would cause an undue burden on family. Some family members would not be able to handle watching a family member deteriorate." | 60 | Black, Non-Hispanic | Male |
| Burden of Dementia | "With dementia, he lives in a state of confusion and fear. This will continue to get worse and quality of life will worsen even more." | 54 | White, Non-Hispanic | Female |
|  | "Dementia doesn't get better, only worse. After a trauma such as a heart attack, the likelihood of any quality of life is very small, and those were his wishes." | 60 | White, Non-Hispanic | Female |
| Religious or Ethical Beliefs | "Life, to all extents, is precious, given by a power greater than the limitations of medical care." | 81 | White, Non-Hispanic | Male |
| Personal Experience | "I lived through that experience with my mother who died from Alzheimer's." | 66 | White, Non-Hispanic | Male |
|  | "I took care of my mom and watched her disappear day by day." | 71 | White, Non-Hispanic | Female |
