# Supplementary material for: Free-Text Responses in a Nationally Representative Experimental Survey about End-of-Life Care Choices: ChatGPT-4o-Assisted Qualitative Analytical Study
Source: JMIR Aging. 2025 Oct 29;8:e76335. doi: 10.2196/76335 (PMC12571202; doi:10.2196/76335)
Supplement: Multimedia Appendix 3 [file aging-v8-e76335-s003.docx]

**Supplementary Table 3. Initial AI generated themes by type of care (comfort care vs. life-extending vs. both) and human verification of AI generated representative quotes with error type**

| Theme/Code | Type of Care | Description | Representative Quote | Verification comments on quotable quotes |
| --- | --- | --- | --- | --- |
| Advanced Directives preferences | Both | Patient’s previous directive on care | “Her wishes according to the directive should be honored.” – 84 White Non-Hispanic Female Adult | Correct |
| Expected Trajectory | Comfort Care | Likely progression of illness leading to the decision | “Her dementia will get worse, so her quality of life will not improve.” - 71 White Non-Hispanic Female Adult | Correct |
| Patient Dignity | Comfort Care | Focus on maintaining dignity in death | “I would want to die with dignity and not be a burden to my family.” | Fabricated, not found in dataset |
| Personal Relationships | Life-Extending Care | Importance of family and relationships | “He would want to spend more time with his family.” | Actual quote: “More time with a loved one is too important” - 41 Hispanic Male with Chronic Disease |
| Caregiver burden | Comfort Care | The burden of care on family and caregivers | “I would not want to continue being a burden on my family.” – 55 White Non-Hispanic Female with Chronic Disease | Correct |
| Prolong lifespan as caregiver duty | Life-Extending Care | Caregivers feel obligated to extend life | “We did everything possible to prolong my father’s life because that’s what he would have wanted.” | Fabricated, but possibly related to the following quote: “Our decision was based on us wanting to keep him with us no matter the state he would be in, or following his wishes. If you do not make the decision you cannot change it later (if vegetative) you just have to make him comfortable while he silently curses us in his head for leaving him that way...lol” 44 Black Non-Hispanic Female Adult |
| Patient Autonomy | Both | Importance of respecting patient decisions | “His previously stated preferences must be respected, even if I disagree.” | Actual quote: “If the person was of sound mind when they made the decision to take extraordinary actions to extend life, even if I disagreed with their decision, I would respect their choice.”- 76 White, Non-Hispanic Male Caregiver |
| Quantity and quality of information | Both | Making decisions based on the amount and clarity of info | “It’s hard to make a decision without knowing the prognosis in detail.” | Actual quote: “Mr. Jones had not expressed any desire for life saving measures versus palliative care before his dementia. Unfortunately, your scenario doesn’t specify what his physical condition would be after the invasive life-saving measures were taken. Would he be awake and aware despite of course, being limited by his dementia? Would he have mobility and the ability to participate in physical activities? Would he be able to eat or would he be forced to use a feeding tube for the rest of his life? It’s really impossible for me to make a decision without knowing these things.” 81 White Non-Hispanic Female Caregiver |
| Religious beliefs and ethics | Life-Extending Care | Ethical or religious reasons for extending life | “Life is precious and should be preserved as long as possible, according to God’s will.” | AI paraphrased. Actual quote: “Life is precious and we should not try to end it of our own accord. It is God’s role to extend or end a life.” - 36 White Non-Hispanic Female Caregiver |
| Emotional strain on family | Comfort Care | Emotional toll of prolonging care | “It’s emotionally draining for the family to keep her alive when she doesn’t even recognize us anymore.” | AI paraphrased: “I have 3 family members with dementia. One is in her 50’s, it was a hard decision I had to make to keep her alive. She is currently in a nursing home and might live for many years. She is healthy but her brain is shrinking, sometimes she can’t recognize family or friends and is unable to speak. I know I made the right decision but she is not doing enjoying life or maybe doesn’t even understand life. I have mixed feelings.” - 54 Hispanic Female Adult |
| Dementia | Comfort Care | Impact of dementia on care decisions | “He doesn’t recognize anyone anymore. Dementia has taken away his quality of life.” | AI paraphrased. Actual quote: “It really depends on what dementia has done to Mr. Jones. If he cannot function as an adult, he cannot ever remember his relatives, then his life quality has diminished. If he can still find joy in some things, and in many ways has become a child, his life quality is undiminished.” 52 White Non-Hispanic Male with Chronic Disease |
| Stages of dementia | Comfort Care | Considering specific dementia stages | “If her dementia is advanced, there’s no point in prolonging life.” | AI paraphrased. Actual quote: “I think she requested to not have life saving treatment. Since she already had advanced dementia I think all the life saving treatment would scare her.” - 43 White Non-Hispanic Female Adult |
| I (the proxy) wouldn’t want to live like this | Comfort Care | Personal belief about life in a similar situation | “I wouldn’t want to be kept alive in this state, so I don’t think it’s fair to put her through it either.” | Correct. |
| Physical function mobility | Life-Extending Care | Preserving mobility and independence | “He still has mobility and can enjoy life in some ways, so extending care makes sense.” | AI combined and paraphrased: “His advanced directive, and the expectation that his physical mobility would be similar.  It stated that he already had mobility issues. He likely already has a routine to live a good life. Even if his mobility is a bit more limited when he gets out it likely wouldn't be much different than it is now.” |
| Limited quality of life with dependence | Comfort Care | Low quality of life due to dependence on others | “Being bedridden and dependent on others is not a life I would want.” | Actual quote: “Would not want to be bedridden or severely dependent on others just to live.” - 69 White Non-Hispanic Male Adult |
| Is invasive treatment worth it? | Comfort Care | Questioning the value of invasive treatment | “Why prolong life with machines if there is no hope of recovery?” | AI paraphrased: “These are tough questions , somethings should be on a case by case basis, but if the only thing that would keep me alive is machines with no chance of getting better, I would just want comfort care.” - 64 White Non-Hispanic Female Adult |
| Financial considerations | Comfort Care | Impact of cost on decision-making | “My father’s treatment was very expensive, and I wouldn’t want my children to go through the same.” | AI paraphrased or fabricated. Original quote that was close in meaning: “Hospitalizations are very expensive, why would anyone choose this hardship on the family when the odds are not in their favor. Why should anyone make their family member suffer in this way. Life is precious but when illness's hit home we must consider the individual that is going through so much pain - 59 Black Non-Hispanic Female Adult” |
| Live a life with value | Life-Extending Care | Belief in preserving life if there is still value | “He still finds value in the small things, like reading and watching TV, so his life is still meaningful.” | AI paraphrased: “What isn't explained is how Mr. Smith FELT about his quality of life AFTER becoming bedridden. Some people adapt and can continue to feel positive about their lives; others cannot. If he was depressed and miserable, do not continue treatments. If he was positive and enjoying talking with friends, playing cards, reading, etc. then do everything to bring him back.” - 75 White Non-Hispanic Female Caregiver |
