# Supplementary material for: Free-Text Responses in a Nationally Representative Experimental Survey about End-of-Life Care Choices: ChatGPT-4o-Assisted Qualitative Analytical Study
Source: JMIR Aging. 2025 Oct 29;8:e76335. doi: 10.2196/76335 (PMC12571202; doi:10.2196/76335)
Supplement: Multimedia Appendix 4 [file aging-v8-e76335-s004.docx]

**Supplementary Material 4. Additional method material to aid in replication of the study.**

**Phase 1: Generating a valid approach**

Prior to initiating the analysis, we conducted a literature review to identify how AI had been used in previous qualitative research. In particular our methods were shaped by articles by Nashwan and Van Veen,[15, 16] who provided prompt examples. Only limited studies have used AI approaches for analyzing free text responses, so we also compared ideas on how to validate our AI findings with other academic teams doing similar work. These discussions yielded several suggestions, including comparing themes side by side between AI and human-generated themes, and doing a keyword count comparison with commonly used qualitative software.

**Phase 2: Familiarization with the data and hand coding**

First, a physician scientist with experience caring for patients at the end-of-life and graduate level qualitative training (EG) and two other qualitatively trained research staff members (MB, JGP) read the first 200 participant responses. Then, two team members (EG, MB) inductively coded the responses to the first 200 responses together. The first 200 responses were chosen because this was sufficient to reach redundancy, or thematic saturation, with the plan to continue should new significant themes continue to emerge.[17, 18] During this session, the team members created a preliminary coding schema in which we identified six major codes and several subcodes relating to end-of-life decision-making (See Supplementary Table 1 for codebook). The codebook was then iteratively refined through group discussion. Then, a third team member independently coded the same 200 responses (JGP). Last, the principal investigator of the study (LHN), who designed the survey and had performed a preliminary qualitative analysis after reading a sample of the responses, provided additional suggestions on codes from her analysis, which we incorporated into the final codebook after team discussion.

**Phase 3: Human-identified themes, subthemes, and illustrative quotes**

Once our codebook reflected the topics and scope of the data from the first 200 respondents, we generated themes and quotes that were illustrative of the major and minor themes. Investigator triangulation (multiple investigators with multiple areas of expertise) was used to establish the trustworthiness of our findings.[19] We compiled a table to display themes and quotes.

**Phase 4: Artificial Intelligence analyses**

Next, we used ChatGPT-4o to extend our analysis by providing it with the entire dataset of 3,931 responders to the free text question. We also piloted other generative AI tools (Microsoft Copilot, ChatGPT-3), but found ChatGPT-4o provided the most usable responses, and allowed us to upload larger amounts of data.

Before entering the dataset in ChatGPT, we cleaned the data by removing all non-responses. Free text containing responses of: “none,” “n/a,” “na,” “no,” “nothing,” “no answer,” or “…”, etc. were removed from the dataset submitted to ChatGPT to limit error. These responses were instead coded as “0” to indicate no free text was available. Data was uploaded via word document containing a table with headings labeled: “Free Text Responses,” “Age,” “Race/Ethnicity,” and “Gender.” ChatGPT processed the entire document at one time and required no chunking of information submission. No API or interface was used.

During the initial upload of the dataset, we provided important context on the task, including that we planned to perform a qualitative analysis of free text responses and that the AI should act as a qualitative analyst using our codebook and data to identify themes and quotable quotes. We also prompted it to find the most common keywords in the text and generate keyword counts.

To illustrate how we completed this analysis, so it can be replicated, we share several sample prompts and how we validated responses.

1. The first prompt set up the research study context, its methods, and the AI’s role. An example prompt is: “This is a large survey study focused on end-of-life decisions, and you are a qualitative researcher analyzing the free text responses to these questions.”
2. Next, we asked ChatGPT-4o to code the data using our codebook. A sample prompt includes: “This is a preliminary coding schema for our research. Using this codebook, code the entire sample. How did you do this?”
3. We asked ChatGPT-4o to create themes and subthemes and identify quotable quotes or key phrases that “make an impression on people from the text”. We then manually searched the dataset in NVivo to ensure the quote was verbatim and not fabricated.
4. Then, we asks the AI to create keywords identified in the entire dataset. We first quantified keywords by querying NVivo using the ‘word frequency/query’ function. NVivo created a list of the most frequently-used words in the dataset with the exception of proper names. NVivo provided the keyword, its frequency in the dataset, and the weighted percentage out of all words in the dataset. Then, we provided each keyword to ChatGPT-4o in addition to supplying it with a data file which included the full dataset of responses. We then queried ChatGPT-4o for exact keyword counts and weighted percentages, e.g. “How often is the word ‘life’ found in this dataset, and what is the weighted percentage of ‘life’ among all words in the dataset?” by asking it to find how many times the specific keyword could be identified in the dataset.
