# Supplementary material for: Free-Text Responses in a Nationally Representative Experimental Survey about End-of-Life Care Choices: ChatGPT-4o-Assisted Qualitative Analytical Study
Source: JMIR Aging. 2025 Oct 29;8:e76335. doi: 10.2196/76335 (PMC12571202; doi:10.2196/76335)
Supplement: Checklist 1 [file aging-v8-e76335-s005.docx]

|  | Item Description | Location (or reason for not reporting) |
| --- | --- | --- |
| **Title & Abstract** |  |  |
| Title | Describe the nature and topic of the study. Identify the study as qualitative or indicate the approach or data collection methods | Top of page 1 |
| Abstract | Summarize the key element of the study using the abstract format of the intended publication | Page 2 |
| **Introduction** |  |  |
| Problem Formulation | Describe the problem/phenomenon studied, its significance, relevant theory and empirical work, and gaps in current knowledge. | Introduction; Paragraphs 1 and 2 |
| Purpose or research  question | Describe the purpose of the study and specific objectives or questions. | Introduction; paragraph 3 |
| **Methods** |  |  |
| Qualitative Approach and Research paradigm | Describe your qualitative approach, your guiding theory (if appropriate), and research paradigm, and reasons for your choices. | Methods; paragraphs 3, 4, and 5 |
| Researcher  characteristics and  reflexivity | Describe how researchers’ characteristics may influence the research, including personal attributes, qualifications/experience, relationship with participants, assumptions, and/or presuppositions;  potential or actual interaction between researchers’ characteristics and the research questions, approach, methods, results and/or transferability. | Methods; paragraph 1 |
| Context | Describe the setting/site(s) in which the study was conducted, why it was selected, and any other salient contextual factors that may influence the study. | Methods; paragraph 1 |
| Sampling Strategy | Describe how and why research participants, documents, or events were selected; criteria for deciding when no further sampling was necessary, and the rationale for those criteria. | Methods; paragraph 1 |
| Ethical issues pertaining to human subjects | Describe any approval by an appropriate ethics review board and participant consent, or explain any lack thereof. Describe any other confidentiality and data security issues. | Methods; paragraph 2 |
| Data collection methods | Describe the types of data collected; details of data collection procedures including (as appropriate) start and stop dates of data collection and analysis, iterative process, triangulation of sources/methods, and modification of procedures in response to evolving study findings. Describe your rationale for these choices. | Methods; paragraphs 1 & 2 |
| Data collections instruments and technologies | Describe any instruments (e.g., interview guides, questionnaires) and devices (e.g., audio recorders) used for data collection; describe if/how the instrument(s) changed over the course of the study. | Methods; paragraph 1 & 2 |
| Units of study | Describe the number and relevant characteristics of participants, documents, or events included in the study. Describe the level of participation. | Methods; paragraphs 1 and 2 |
| Data processing | Describe the methods for processing data prior to and during analysis, including transcription, data entry, data management and security, verification of data integrity, data coding, and anonymisation / deidentification of excerpts. | Methods; Paragraphs 3, 4, 6, and 8 |
| Data analysis | Describe the process by which inferences, themes, etc. were identified and developed, including the researchers involved in data analysis; usually references a specific paradigm or approach. Describe why you chose this process. | Methods; Paragraphs 6 and 7 |
| Technique to enhance trustworthiness | Describe any techniques to enhance trustworthiness and credibility of data analysis,(e.g., member checking, triangulation, audit trail). Describe why you chose these techniques. | Methods; Paragraph 7 |
| Results |  |  |
| Links to empirical data | Provide evidence (e.g., quotes, field notes, text excerpts, photographs) to substantiate analytic findings. | Tables 2, 3, and 5 |
| Discussion |  |  |
| Integration with prior work, implications, transferability, and contribution(s) to the field | Summarize the main findings, explain how findings and conclusions connect to, support, elaborate on, or challenge conclusions of earlier scholarship; discuss the scope of application/generalizability; identify unique contribution(s) to scholarship in a discipline or field. | Discussion; paragraphs 2, 3, 4, 5, 6, and 7 |
| Limitations | Discuss the trustworthiness and limitations of findings | Page 16 |
| Other |  |  |
| Conflict of interests | Describe any potential sources of influence or perceived influence on study conduct and conclusions. Describe how these were managed. | Page 17 |
| Funding | Describe sources of funding and other support. Describe the role of funders in data collection, interpretation, and reporting. | Page 17 |
